# Tumor Microenvironment and Microvascular Density in Human Glioblastoma

**DOI:** 10.3390/cells12010011

**Published:** 2022-12-20

**Authors:** Roberto Tamma, Giuseppe Ingravallo, Tiziana Annese, Antonio d’Amati, Loredana Lorusso, Domenico Ribatti

**Affiliations:** 1 Department of Translational Biomedicine and Neuroscience, University of Bari Medical School, 70124 Bari, Italy; 2 Department of Precision and Regenerative Medicine and Ionian Area, University of Bari Medical School, 70124 Bari, Italy; 3 Department of Medicine and Surgery, Libera Università del Mediterraneo (LUM) Giuseppe Degennaro University, 70010 Bari, Italy

**Keywords:** angiogenesis, BCL-6, glioblastoma, lymphocytes, macrophages, tumor microenvironment

## Abstract

Glioblastoma (GBM) is a very aggressive form of cancer affecting the central nervous system. Although it occurs almost exclusively in the brain, glioblastoma can also appear in the brainstem, cerebellum, and spinal cord. It is characterized by high rates of proliferation, invasion, and necrosis. Moreover, GBM is a highly vascularized tumor and presents resistance to therapy. Recent data indicate that GBM cells are surrounded by a microenvironment (TME) which includes a complex network constituted of cellular/extracellular components and vessels able to influence both tumor growth and angiogenesis. In this retrospective study, we evaluated 30 bioptic specimens of adult patients diagnosed with IDH1 wild type GBM taken at the time of the first diagnosis. Each section has been divided into two experimental zones: the tumor side and the healthy surrounding tissue. We performed a series of immunohistochemical stainings with the purpose of evaluating the presence of total and M2 macrophages, CD4^+^-, CD8^+^-lymphocytes, and CD34^+^ microvessels. In addition, we have also evaluated the percentage of cells expressing bcl6 and p53 to determine any possible correlations with TME. Our data showed a significant increase in the total and M2 type macrophages, of CD4^+^ and CD8^+^ lymphocytes, and of CD34^+^ microvessels in the tumoral area respective to the healthy zone. We also confirmed our previous data showing the higher number of p53 and BCL6^+^ cells in the tumor area with a positive correlation between BCL6 and CD34^+^ microvessels. In conclusion, the data that came from this work support the important role played by microenvironment components in GBM progression. These results could contribute to the generation of new specific therapies useful in preventing GBM progression.

## 1. Introduction

Primary cancerous tumors of the brain are the 17th most numerous cancer type worldwide and approximately 77% of these are gliomas [1,2,3]. Glioblastoma multiforme (GBM), a high-grade glioma classified as IDH mutated WHO Grade 4, is a very diffuse and aggressive primary brain tumor in adults [4]. The GBM characteristics are intense proliferation, tendency to spread, induction of necrosis, high microvascular density, and resistance to treatment [5]. The GBM standard treatment includes surgery, associated with radio- and chemotherapy combination, or the use of alternating electrical fields, which expand median overall survival to 21 months [6,7,8]. The high heterogeneity of GBM is due to its tumor microenvironment (TME). The GBM TME is constituted of malignant astrocytoma cells and cancer stem cells (CSCs), immune cells, stromal cells, endothelial cells, and pericytes, creating separate niches within the tumor and neurons [9,10]. The GBM TME has acquired a crucial role in better understanding the intratumor heterogeneity [11], its resistance to conventional and emerging treatments [12], or immune escape [13]. Its TME is extremely characteristic and scarcely accessible, and thus a GBM result hard to treat. Up to more than a third of the GBM mass can be constituted especially of myeloid-derived cells, and in particular of macrophages [14], whereas in healthy conditions, the amount of immune infiltrating cells in the brain is very low due to being restrained by the blood-brain barrier (BBB) [14]. The latter is hampered in GBM because of abnormally organized blood vessels and their reduced structural integrity, which led to increased interstitial fluid pressure, hypoxia, necrosis, as well as edema. [15]. GBM-associated macrophages are recruited by tumor-derived signals [16] and it is believed that these are involved in mediating immune suppression and promoting invasion [3]. The TME determines if macrophages will assume the M1 and/or M2 phenotype [17]. Infiltrating macrophages represent a negative prognostic factor for survival in murine models of high-grade gliomas [18,19,20]. Tumor-infiltrating lymphocytes (TILs) have the potential to exert both pro- and antitumor effects in TME. In many cancers, they have also been associated with the prognostic index, however, their role in GBM has not been fully elucidated. It is known that both CD4^+^ and CD8^+^ lymphocytes, likewise T helper, FoxP3^+^ Treg, myeloid suppressor cells (MDSCs), and natural killer (NK) cells, infiltrate GBM [21]. CD8^+^ T-lymphocytes are crucial for tumor clearance while representing less than a quarter of TILs [22]. These cells are thought to have exhausted phenotypes and compromised effector capabilities, making them useless in their function as cytotoxic lymphocytes. [23]. Likewise, CD4^+^ T-lymphocytes may correlate with poor survival outcomes [24]. IDO expression is induced by tumor-infiltrating T cells in GBM, contributing to decreased patient survival [25]. Moreover, CLOK is involved in tumor progression on GBM through an inflammatory pathway [26]. Due to the inherent and extrinsic characteristics of the tumor, GBM TME has an immunosuppressive effect [27]. This immunosuppressive environment presents the proper treatment of this cancer. In fact, many therapies successfully used for immunogenic tumors have failed against GBM [28]. Therefore, novel therapies are urgently needed. However, for ideal drug development, it is essential to deepen the TME knowledge to better understand its role in GBM. A novel idea in the treatment of cancer is epigenetic therapy. It works by altering the expression of numerous genes linked to the development of cancer, which may have antineoplastic effects [29]. A frequent genetic alteration seen in primary central nervous system lymphoma is Bcl-6 translocation [30] although uncertainty about its function in brain malignancies. Previous studies demonstrated a correlation between Bcl-6 translocation frequency, expression in GBM patients, and the disease’s severity. [31]. BCL-6 overexpression inhibits caspase-3 expression and apoptotic process. Additionally, GBM showed higher levels of p53 expression as well as observed for Bcl-6 [31]. 

In this retrospective study, we evaluated by immunohistochemical analysis the cell content of GBM TME and microvessels, in both the tumoral area and outside it. Specifically, we calculated the proportions of CD4^+^ and CD8^+^ lymphocytes, total and M2 macrophages, and CD34^+^ vessels. Finally, we calculated the proportion of cells that express p53 and bcl6 to look for any associations with TME.

## 2. Materials and Methods

### 2.1. Patients

This retrospective study included bioptic specimens derived from 30 adult patients diagnosed with IDH1 wild type GBM taken at the time of first diagnosis. Each section has been divided into two experimental zones: the tumor side (GBM) and the healthy surrounding tissue (CTRL). The samples were collected from the archive of the Section of Pathology of the University of Bari School of Medicine, Italy, between 2019 and 2021. All procedures were in accordance with the ethical standards of the responsible committee on human experimentation (institutional and national) and with the Helsinki Declaration of 1964, and later versions and signed informed consent from individual patients were obtained to conduct the study. 

### 2.2. CD4, CD8, CD68, CD163, CD34, Ki67, Bcl6 and p53 Immunohistochemistry

Serial histological sections of 4 um thickness, collected on poly-L-lysine-coated slides (Sigma Chemical, St Louis, MO, USA), were deparaffinized. The sections were rehydrated in a xylene-graded alcohol scale and then rinsed for 10 min in 0.1 M PBS. Sections were pretreated with sodium citrate pH 6.1 solution (DAKO, Glostrup, Denmark) for antigen retrieval for 30 min at 98 °C and then incubated with mouse monoclonal anti-CD4 (DAKO, Glostrup, Denmark), mouse monoclonal anti-CD8 (DAKO, Glostrup, Denmark), mouse monoclonal anti-CD68 (DAKO, Glostrup, Denmark), mouse monoclonal anti-CD163 (DAKO, Glostrup, Denmark), mouse monoclonal anti-CD34 (DAKO, Glostrup, Denmark), mouse monoclonal anti-Ki67 (DAKO, Glostrup, Denmark), monoclonal anti-bcl6 (DAKO, Glostrup, Denmark) and monoclonal anti-p53 (DAKO, Glostrup, Denmark) diluted 1:50, 1:50 1:100, 1:100, 1:100, 1:50, 1:100, and 1:100 respectively. Thereafter, the sections were counterstained with Mayer hematoxylin and mounted in synthetic medium. Specific preimmune serum (DAKO), replacing the primary antibodies, served as negative control. The sections from each experimental group were scanned using the whole-slide morphometric analysis scanning platform Aperio Scanscope CS (Leica Biosystems, Nussloch, Germany). All the slides were scanned at the maximum available magnification (40×) and stored as digital high-resolution images on the workstation associated with the instrument. Digital slides were inspected with Aperio ImageScope v.11 software (Leica Biosystems, Nussloch, Germany) at 20× magnification, and 10 fields with an equal area were selected for the analysis at 40× magnification in both the center of the tumor and in the healthy surrounding areas. The protein expression was assessed with the Positive Pixel Count algorithm embedded in the Aperio ImageScope software and reported as positivity percentage, defined as the number of positively stained pixels on the total pixels in the image. 

### 2.3. Statistical Analysis

Data derived from the center of a GBM tumor (GBM) and from the healthy surrounding areas (CTRL) are reported as means ± SE. All variables were checked for normality (Shapiro-Wilk normality test) to see the population distribution. Unpaired two tailed *t*-test for mean values and Pearson’s correlation coefficient for linear regression analysis were used to compare parameters with normal distribution. Data are presented as scatter dot plots with mean and standard error, with all data points shown. The Graph Pad Prism 5.0 statistical package (GraphPad Software, San Diego, CA, USA) was used for analyses and the limit for statistical significance was set at *p* < 0.05. 

## 3. Results

### 3.1. CD68 and CD163 Immunohistochemistry

**Figure 1:**
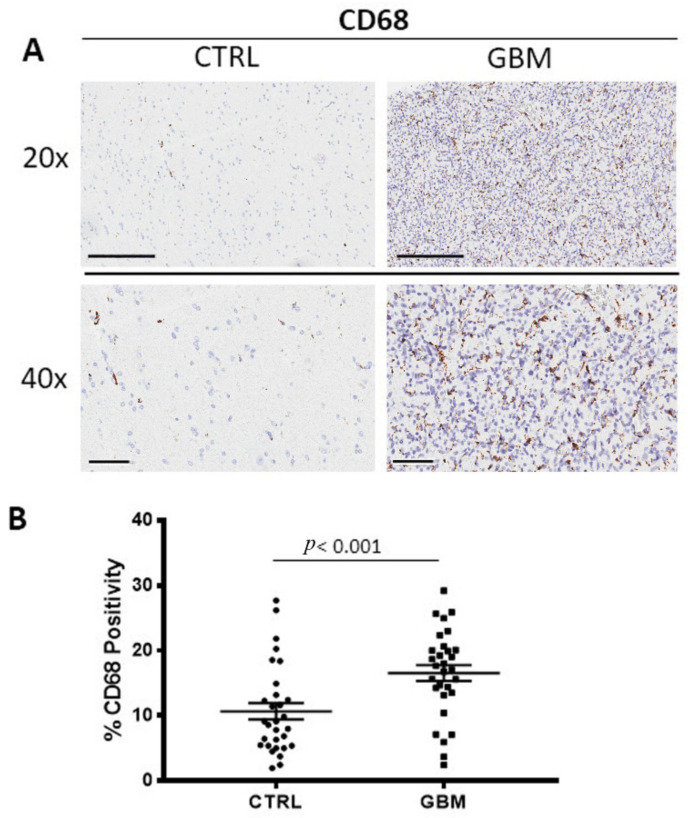
(**A**) Immunohistochemical staining for CD68 in order to identify the total macrophages in both the center of tumor (GBM) and in the healthy surrounding areas (CTRL). Scale bar: 60 μm (40×), 200 μm (20×). (**B**) Morphometric analysis indicating the percentage of CD68 positivity in GBM and CTRL samples.

**Figure 2:**
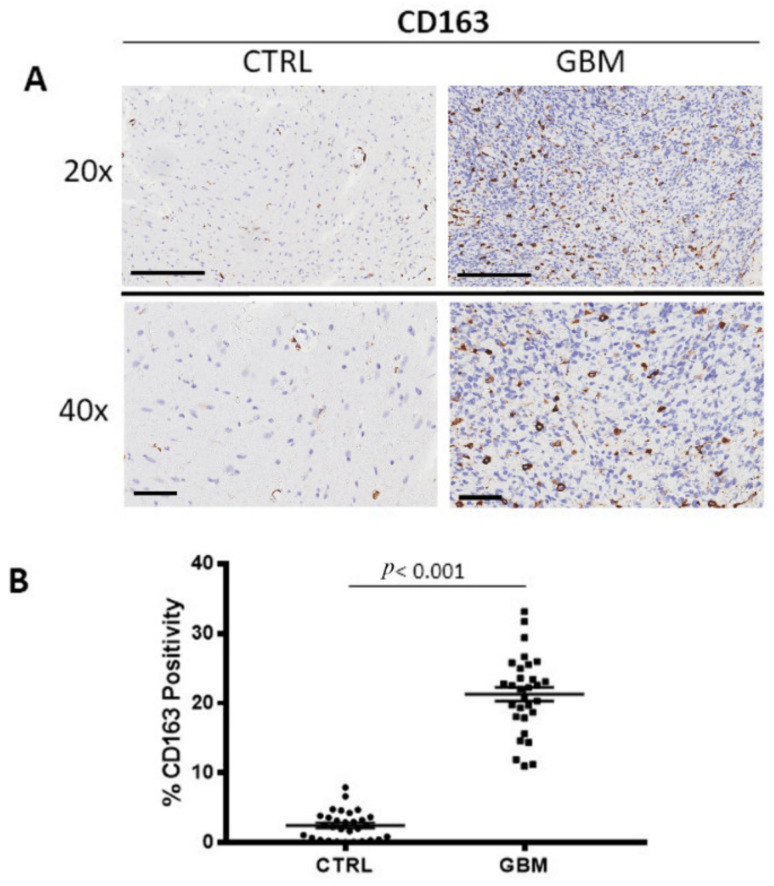
(**A**) Immunohistochemical staining for CD163 as a marker of M2 macrophages in both the center of tumor (GBM) and in the healthy surrounding areas (CTRL). Scale bar: 60 μm (40×), 200 μm (20×). (**B**) Morphometric analysis indicating the percentage of CD163 positivity in GBM and CTRL samples (**B**).

GBM tissue samples were subjected to immunohistochemistry for CD68 (Figure 1A) and CD163 (Figure 2A) in order to evaluate total and M2 macrophages, respectively, in both the center side of the tumor and in the healthy surrounding areas. Morphometric analysis (Figure 1B and Figure 2B) evidences the significantly increased numbers of CD68+ and CD163+ cells in the center of GBM tumors (CD68:16.53 ± 1.2%; CD163: 21.28 ± 0.4%) as compared to the CTRL (CD68: 10.66 ± 1.2%; CD163: 2.5 ± 1%). 

### 3.2. CD4 and CD8 Immunohistochemistry

**Figure 3:**
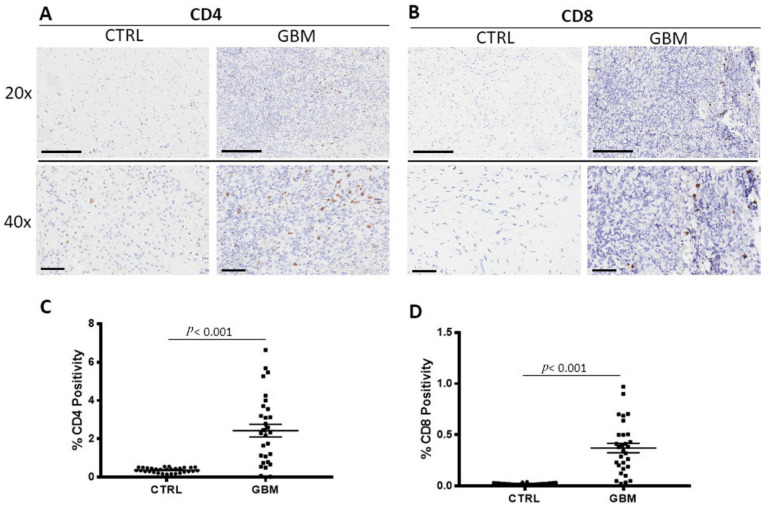
Immunohistochemical staining for T- helper lymphocytes CD4^+^ (**A**) and cytotoxic T- lymphocytes CD8^+^ (**B**) in both the center of tumor (GBM) and in the healthy surrounding areas (CTRL). Scale bar: 60 μm (40×), 200 μm (20×) (**A**). Morphometric analysis indicating the percentage of CD4 (**C**) and CD8 (**D**) positivity in GBM and CTRL samples.

GBM samples were immune stained for CD4 and CD8 to evaluate CD4 (Figure 3A) and CD8^+^ lymphocytes (Figure 3B) in both the center of the tumor and in the healthy surrounding areas. Morphometric analysis (Figure 3C,D) indicated the significant increase of CD4^+^ and CD8^+^ lymphocytes density in the tumor side GBM (CD4^+^: 2.4 ± 0.33%, CD8^+^: 0.24 ± 0.04%), as compared to the CTRL (CD4^+^: 0.4 ± 0.02%; CD8^+^: 0.02 ± 0.001%).

### 3.3. CD34 Immunohistochemistry

**Figure 4:**
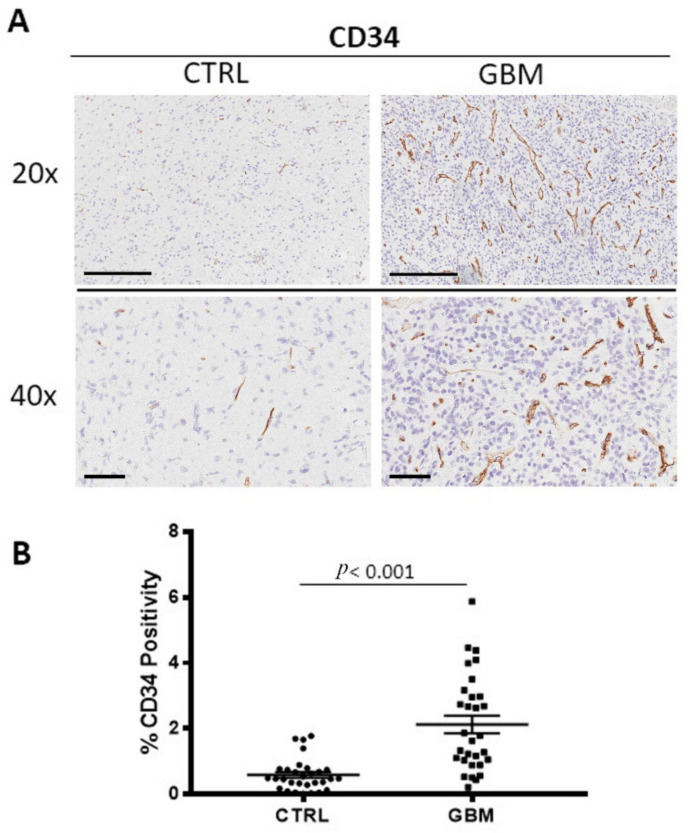
(**A**) Immunohistochemical staining for CD34 microvessels in both the center of tumor (GBM) and in the healthy surrounding areas (CTRL). Scale bar: 60 μm (40×), 200 μm (20×). (**B**) Morphometric analysis indicating the percentage of CD34 microvessel positivity in GBM and CTRL samples (**B**).

GBM specimens were immune stained for CD34 (Figure 4A), to estimate the microvessel density in the tumor and in healthy zone. Morphometric analysis (Figure 4B) showed the significant increase of CD34+ into the tumor tissue (CD34: 2.1 ± 0.3%) as compared to the healthy tissue, CTRL (CD34: 0.58 ± 0.09%). 

### 3.4. Ki67, Bcl6 and p53 Immunohistochemistry

**Figure 5:**
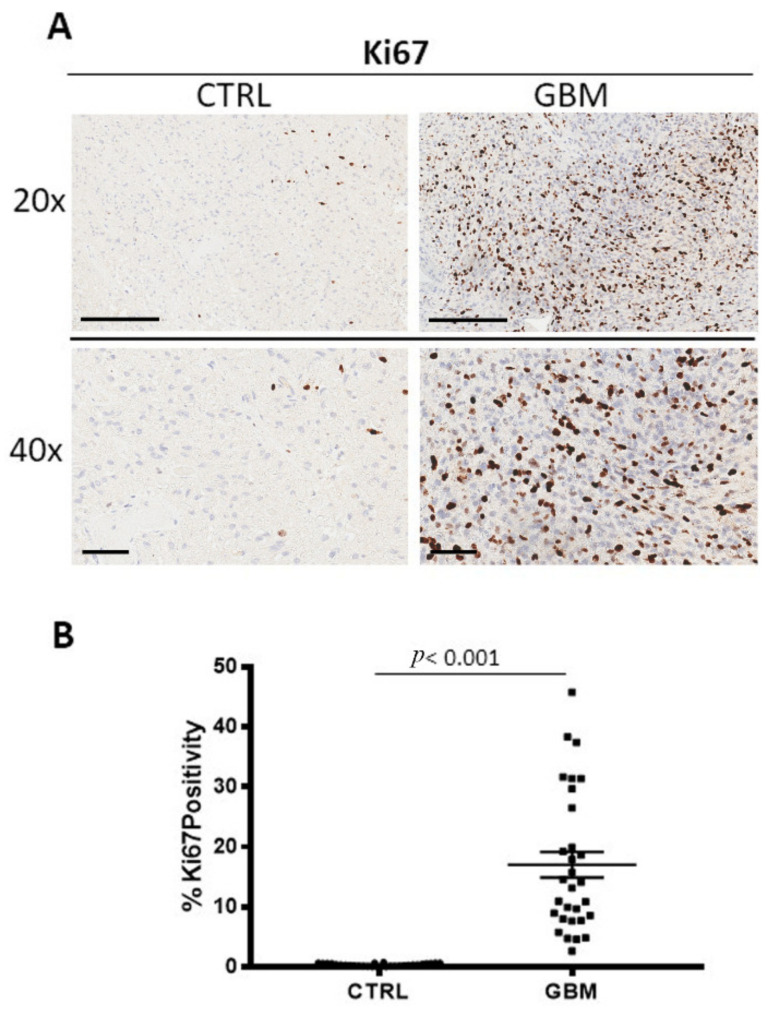
(**A**) Immunohistochemical staining for Ki67 as marker of proliferation in both the center of tumor (GBM) and in the healthy surrounding areas (CTRL). Scale bar: 60 μm (40×), 200 μm (20×). (**B**) Morphometric analysis indicating the percentage of Ki67 positivity in GBM and CTRL samples.

**Figure 6:**
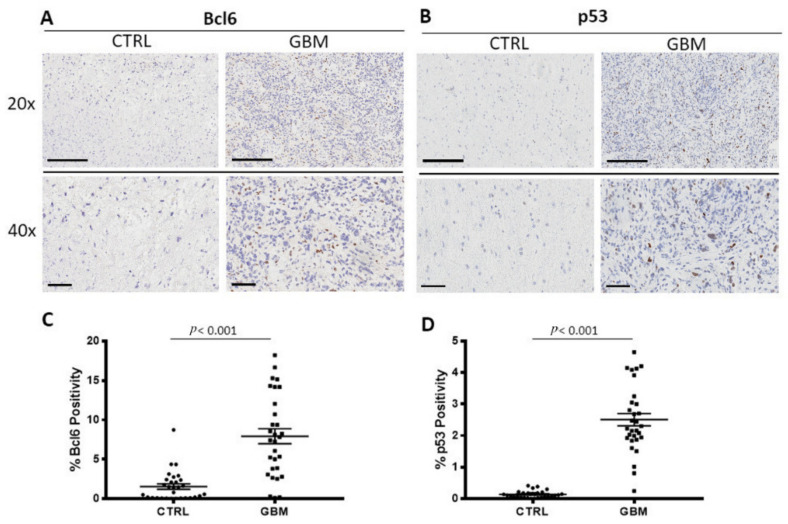
Immunohistochemical staining for Bcl6 (**A**) and p53 (**B**) in both the center of tumor (GBM) and in the healthy surrounding areas (CTRL). Scale bar: 60 μm (40×), 200 μm (20×) (**A**). Morphometric analysis indicating the percentage of BCL6 (**C**) and p53 (**D**) positivity in GBM and CTRL samples.

GBM specimens were immune stained Ki67 (Figure 5A), Bcl6, and p53 (Figure 6A,B) to evaluate the proliferating cells, the Bcl6, and p53 positive cells, respectively. Morphometric analysis (Figure 5B), (Figure 6C,D) showed the significant increase of Ki67 positive cells in the tumor tissue (Ki67: GBM (14.04 ± 2.1%)) as compared to the healthy tissue, CTRL (Ki67: 0.38 ± 0.04%). As concerns Bcl6 and p53, we observed the significant increase of both in the tumor (Bcl6: GBM (7.9 ± 0.98%), p53: GBM (2.5 ± 0.2%)) with respect to the surrounding healthy area (Bcl6: CTRL (1.55 ± 0.35%), p53: CTRL (0.14 ± 0.02%)).

### 3.5. Correlation Analysis 

**Figure 7:**
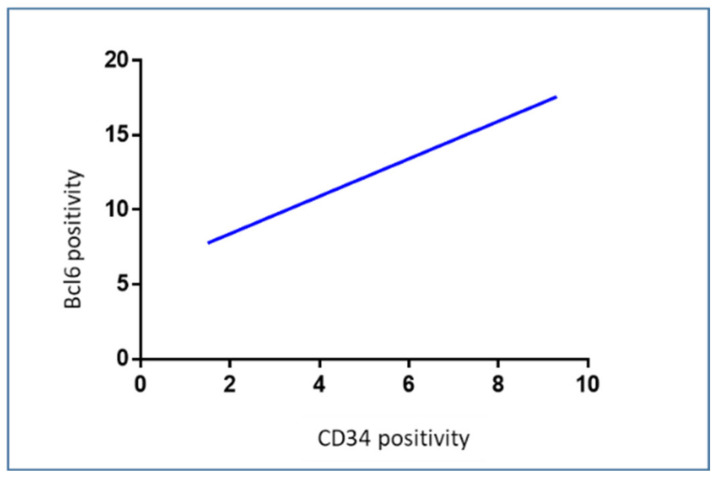
Regression analysis graph indicating the correlation between Bcl6 and CD34 in GBM samples (rho = 0.58, *p* = 0.002).

A positive correlation between Bcl6 and CD34 (rho = 0.58, *p* = 0.002), was found by Pearson’s correlation analysis (Figure 7).

## 4. Discussion

The most prevalent and dangerous primary brain tumor in adults is GBM. The treatments include surgical resection, radiochemotherapy, and adjuvant chemotherapy. The median survival ranges from nine to twenty-one months [7,32]. Its lethality is caused by the tumor cells’ fast proliferation and ongoing invasion of neighboring healthy brain tissue. [33]. It should be considered that extensive brainstem infiltration, and not mass effect, is a common feature of end-stage cerebral GBM [34]. The identification of biomarkers would be useful for both prognosis and therapeutic purposes. For several cancers, blocking immune checkpoint signaling pathways such as the PD-1/PD-L1 axis and CTLA4 significantly improves survival [35,36]. The outcomes of the current phase II/III randomized study NRG-BN007, which compares temozolomide to radiation in patients with unmethylated GBM, are being eagerly awaited [37]. The survival advantage of immunotherapy and targeted therapy has not been established [38,39], and one of the reasons is the particular microenvironment of GBM [40,41,42]. This makes it necessary to determine new treatment options for GBM. The tumor microenvironment (TME) is a complex environment that surrounds cancer cells [43] and consists of cellular, extracellular, and vascular components that are also important in determining therapy success [44]. One of the most important hallmarks of GBM is the tumor heterogeneity at the inter- and intratumor levels. This diversity is further enhanced by interactions among the many GBM microenvironment elements, which significantly contribute to the development of the disease. [45]. The GBM microenvironment contains other cell types in addition to neoplastic tumor cells, vascular cells, and immune cells. TME specifically consists of immune cells, central nervous system (CNS) resident cells, glioma stem cells (GSCs), fibroblasts, endothelial cells, and pericytes. Tumor-associated macrophages (TAMs) in GBM consist in two distinct macrophage populations, the bone marrow derived macrophages, and microglia [46,47] although they were not histologically distinguished. Scientific literature data show that microglia and macrophages play primarily a protumorigenic role [48,49] although antitumoral effects were also described [50]. We analyzed and compared the area inside and outside the GBM tissue, the latter characterized by a very high percentage of Ki67^+^ cells, evaluating the CD68^+^ (total) and CD163^+^ (M2) macrophages positivity. Our results indicate a significant increased number of both CD68^+^ and CD163^+^ cells in the center of the GBM tumor with respect to the outside. In particular, the presence of CD163^+^ cells outside the GBM tumor tissue were very low. These results indicate the important role of the CD163^+^ macrophage population in GBM progression. In fact, as widely described in scientific works, the M2 subset enhances immunosuppression and angiogenesis in tumor progression. In relation to angiogenesis, GBM is characterized as very vascularized and from our evaluations, the quantity of CD34^+^ cells in the tumor mass resulted in times greater than in surrounding tissue. 

It has been shown that tumor-infiltrating lymphocytes (TILs) play a prognostic and predictive function in many cancers. [51]. Although a positive correlation between TILs and overall survival has been found in many cancers, their function in GBM is still debatable, and it is yet unknown how distinct T-cell subsets contribute to the orchestration of a tumor-specific immune response. [52,53]. High CD4^+^ cells infiltrate is associated with poor prognosis because it shifted the tumor cytokine milieu towards immunosuppression, preventing immune destruction of tumor cells [54].

Intratumoral densities of proliferating CD8^+^ cells and higher CD8^+^/CD4^+^ ratios are independent predictors of overall survival in patients with GBM [55]. The samples evaluated in this work showed a significant increase of both CD4^+^ and CD8^+^ cells in the tumor zone with respect to the surrounding area although the percentage increase of CD4^+^ cells was about ten times greater than that observed in CD8^+^ indicating the unbalance of their ratio resulting in its decrease.

The transcriptional repressor Bcl6, which is essential for the growth and activities of B and T cells, is encoded by the proto-oncogene BCL6. [56]. Moreover, Bcl6 is involved in the regulation of cell proliferation and apoptosis [57]. The BCL6 protein has been found expressed in solid malignancies [58], and in most cases, its expression is associated with poor prognosis and worse outcomes [59]. Previously, we demonstrated a frequency of 36.6% of Bcl-6 translocation in GBM IDH1 mutated patients in association with highly expression of Bcl-6 at protein and messenger levels, demonstrating that Bcl-6 plays a role in the repression of apoptosis of cancer cells and its expression correlated with p53 [31]. The mutant form of p53 is overexpressed by many malignant tumors, including lymphomas and GBM [60]. These relatively understudied p53 mutants promote GBM malignancy through the activation of genes other than those regulated by wild type p53. This leads to higher proliferation, invasion, and a more stem-like phenotype [61,62]. Previously, we have found the p53 gene strongly expressed in GBM in correlation with Bcl-6 expression [31]. In this study, we observed the increased number of p53^+^ cells in the tumor mass with respect to the control zone but we did not find any correlation with the TME. As we reported previously, probably p53 is not functional since it is mutated or because the BCL6 effect is predominant in GBM. As regards BCL6, the number of positive cells also significantly increased in the tumor area and this data positively correlated to the CD34-positive vessels indicating a direct or indirect role of BCL6 in angiogenesis, as discussed in our previous work [31], however, this would need further investigation.

## 5. Conclusions

The findings of this study support the significance of tumor microenvironment elements, such as M2 macrophages, the balance of CD8^+^ and CD4^+^ lymphocytes, microvascular density, and epigenetic events, such as BCL6 translocation and p53 expression, in the development of GBM. We confirmed an elevated proportion of p53- and BCL6-positive cells in the GBM tumor region, with the latter finding as a positive correlation with CD34-positive microvessels. Overall, these data confirm that the presence of immune and inflammatory cells contribute to modulating tumor growth and invasion in GBM. In this context, as we have analyzed in this work, morphological and morphometric investigation of the different cellular components of the tumor microenvironment in GBM is an important aspect in the assessment of the progression of this human tumor. Finally, these findings might aid in the identification of novel, focused treatments targeting inflammatory cells that can reduce the progression and spread of GBM.

## Data Availability

The data used to support the findings of this study are available upon request to the authors.
